# Emerging Roles for the Orphan GPCRs, GPR37 and GPR37 L1, in Stroke Pathophysiology

**DOI:** 10.3390/ijms23074028

**Published:** 2022-04-05

**Authors:** Sabra Mouhi, Breona Martin, Sharon Owino

**Affiliations:** Neuroscience Program, Smith College, Northampton, MA 01063, USA; smouhi@smith.edu (S.M.); bmartin@smith.edu (B.M.)

**Keywords:** ischemic stroke, GPR37, GPR37 L1, inflammation, gliosis, astrocytes, microglia, NPC

## Abstract

Recent studies have shed light on the diverse and complex roles of G-protein coupled receptors (GPCRs) in the pathophysiology of stroke. These receptors constitute a large family of seven transmembrane-spanning proteins that play an intricate role in cellular communication mechanisms which drive both tissue injury and repair following ischemic stroke. Orphan GPCRs represent a unique sub-class of GPCRs for which no natural ligands have been found. Interestingly, the majority of these receptors are expressed within the central nervous system where they represent a largely untapped resource for the treatment of neurological diseases. The focus of this review will thus be on the emerging roles of two brain-expressed orphan GPCRs, GPR37 and GPR37 L1, in regulating various cellular and molecular processes underlying ischemic stroke.

## 1. Introduction

Stroke is currently the second leading cause of death worldwide with an annual mortality rate of approximately 5.5 million [1]. Ischemia accounts for greater than 85% of stroke incidents, with the remaining 15% classified as being hemorrhagic in nature [2]. Immediately following ischemic stroke, patients may experience loss of vision, impaired speech, and/or paralysis as a result of disrupted blood flow most commonly caused by thrombosis or embolism [3]. More than 50% of stroke patients suffer chronic disabilities and cognitive deficits due to the lack of effective treatments available to promote recovery and repair [4]. The diversity of pathological processes which occur during both the acute and chronic phases following ischemic injury create exceptional challenges for therapeutic strategies targeting stroke [5].

Currently, tissue plasminogen activator (tPA) is the only FDA-approved treatment for reestablishing blood flow and mitigating tissue damage following stroke [6]. However, tPA is used in less than 10% of stroke patients due to the fact that this treatment must be delivered within a very narrow time window (3–4.5 h) [6]. A comprehensive understanding of the complex pathophysiology of stroke in both time and space is needed to uncover novel therapeutic targets. Recent studies have shed light on the diverse roles of G-protein coupled receptors (GPCRs) on the pathophysiology of stroke. These receptors constitute a family of seven transmembrane-spanning proteins that play an intricate role in cellular communication by binding to a diverse array of ligands, such as protons, lipids, neurotransmitters, and light [7].

A number of GPCRs within the central nervous system (CNS) have been found to play a significant role in the mechanisms underlying the stroke response [8]. Notably, dopamine 2 receptors (D2R) have been implicated in playing a generally protective role following ischemic damage by regulating glial cell responses, inflammation, and ecotoxicity [9,10,11]. In contrast, the activation of Adenosine A_2A_ receptors (A_2A_R) appears to be more complex and mediate both beneficial and detrimental effects following ischemic injury [12,13,14]. A number of other Class A GPCRs such as endothelin receptors, serotonin receptors, metabotropic glutamate receptors, and prostaglandin receptors have also been highlighted as mediators of stroke pathophysiology [8]. Much less is known about the contribution of orphan GPCRs to the pathological processes underlying stroke progression. This unique class of GPCRs, for which no natural ligands have been found, offers vast therapeutic potential for drug development. Interestingly, the majority of these receptors are expressed within the CNS, therefore providing untapped opportunities for the treatment of neurological diseases [15]. The focus of this review will thus be on the roles of two brain expressed orphan GPCRs, namely GPR37 and GPR37L1, in regulating various pathological processes underlying ischemic stroke.

### 1.1. Structure and Tissue Expression

**Figure 1:**
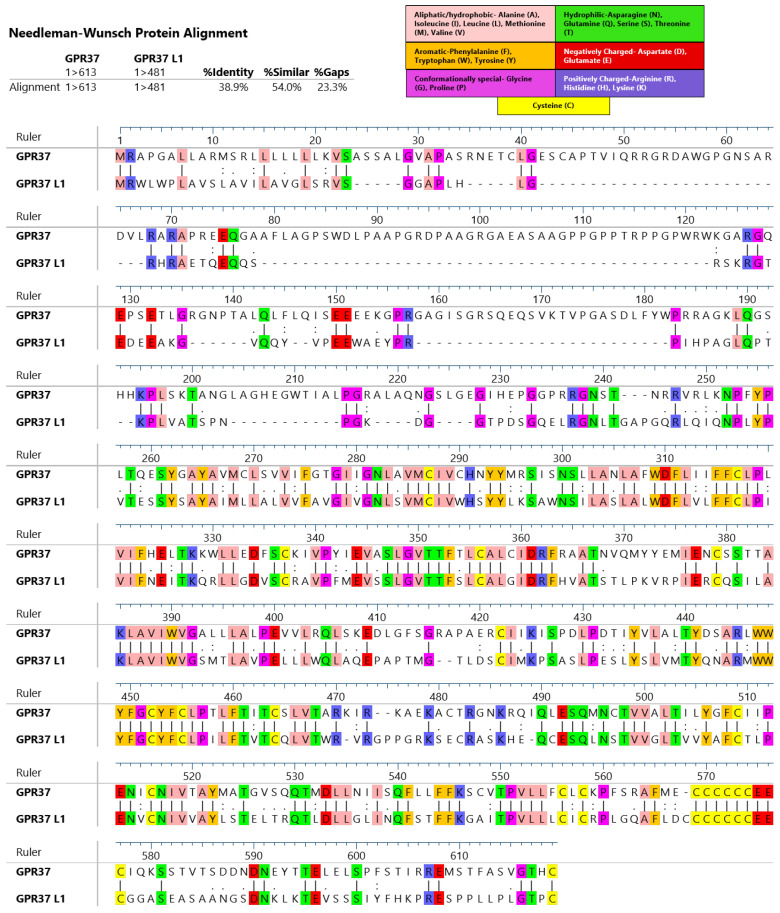
Alignment of human GPR37 and GPR37L1 primary structure using pairwise alignment. Residues with conserved sequences are highlighted by color coding which classifies these residues according to their physiochemical properties. Alignment results were obtained and the image was created using Lasergene MegAlign Pro software from DNASTAR, Inc (Madison, WI, USA).

GPR37 was discovered in the late 1990s through the expressed sequence tag analysis of a human hippocampal library [16]. The receptor was initially named endothelin B receptor-like protein (ETBR-LP) due to its high sequence homology to endothelin receptor B (ET_B_), with which it shared 52% similarity and 27% identity [16]. One year following the discovery of GPR37, GPR37 L1 (at the time named ETBR-LP-2) was identified from a screen using samples obtained from human caudate nuclei [17]. GPR37L1 holds 54% similarity and 39% identity to GPR37 (Figure 1). Within both humans and mice, the genetic locus for GPR37 L1 is located on chromosome 1. In contrast, GPR37 is located on human chromosome 7 and mouse chromosome 6 [18].

Both GPR37 and GPR37 L1 are almost exclusively expressed within the CNS, although with rather different glial expression patterns. GPR37 is predominantly found in late-stage (pre-myelinating and myelinating) oligodendrocytes, but not expressed within oligodendrocyte progenitor cells (OPCs) [19]. GPR37 is also expressed by specific neuronal subsets such as dopaminergic neurons of the substantia nigra [20], hippocampal neurons [21], as well as the optic nerve and spinal cord [21]. The expression of GPR37 has also been noted within neural progenitor cells (NPCs) [22,23] and peripheral macrophages [24]. In contrast to GPR37, GPR37 L1 is highly enriched within astrocytes and OPCs [19,25] as well as quiescent NPCs within the subventricular zone of the lateral ventricle (SVZ) [26].

### 1.2. Current Proposed Ligands

Since both GPR37 and GPR37L1 exhibit strong sequence similarity to endothelin receptors and other peptide-activated GPCRs [27], it has been suspected that these receptors are activated via an endogenous peptide ligand. One of the earliest proposed ligands for GPR37 was the invertebrate peptide head activator (HA) [28,29]. These early studies demonstrated that HA stimulated receptor internalization and intracellular calcium accumulation within heterologous cells overexpressing GPR37 [29]. Similarly, HA was also reported to trigger the calcium-mediated nuclear factor of activated T-cell reporter (NFAT) gene transcription and to inhibit adenylyl cyclase activity within HEK293T cells overexpressing GPR37 [30]. To date, a number of studies have failed to find evidence supporting HA-mediated receptor activation [31,32]. However, by far the most compelling data against HA being a potential ligand for GPR37 has been its lack of expression within the human genome [33].

More recent studies have demonstrated that the protein prosaposin and its peptide fragment prosaptide (whose synthetic analog is TX14A) induce receptor internalization, ERK1/2 phosphorylation, and the inhibition of forskolin stimulated cAMP levels in heterologous cells overexpressing GPR37 or GPR37 L1 [34]. These seminal studies also demonstrated the ERK1/2-mediated activation of both receptors via TX14A within primary and astrocytes [34] and concluded that both GPR37 and GPR37L1 were Gαi-coupled receptors. Notably, prosaposin is upregulated in animal models of ischemic stroke [35,36] and generally thought to be functioning in a neuroprotective manner [37]. A recent study highlighting the role of GPR37 in mediating glial cell responses following stroke examined prosaposin levels within the peri-infarct region of wild-type and GPR37 knock-out (*Gpr37^-/-^*) mice and did not observe detectable differences in prosaposin expression [23]. Similar to studies proposing HA as the endogenous ligand for GPR37, there have been a number of studies which have failed to recapitulate specific prosaposin/prosaptide-mediated effects on GPR37/GPR37 L1 signaling [32,38,39]. It has been posited that the conflicting findings being presented by various groups could specifically relate to the cellular context in which the receptor was studied [40]. The latter group observed TX41A mediated activation and protection from oxidative stress via GPR37 and GPR37 L1 within primary astrocytes, but failed to observe TX41A mediated activation within heterologous cells overexpressing either receptor construct [40]. This discrepancy could suggest that the activation of GPR37 and GPR37 L1 is dependent on the expression of cell-specific scaffolding proteins or interacting partners.

**Figure 2:**
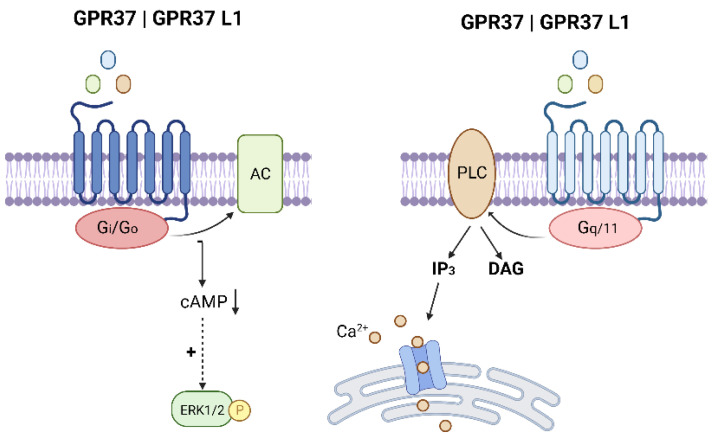
Proposed downstream signaling pathways for GPR37 and GPR37 L1. (Gi/o = G alpha i/o subunit; AC = adenylyl cyclase; cAMP = cyclic AMP; ERK = extracellular signal-regulated kinase, P = phosphate; PLC = phospholipase C; Gq/11 = G alpha q/11 subunit; IP_3_ = inositol trisphosphate; DAG = diacylglycerol, Ca^2+^ = calcium). Image created with BioRender.com (accessed on 26 March 2022).

Additional studies have proposed two subsequent ligands for GPR37: the bioactive lipid metabolite neuroprotectin D1(NPD1) [24] and the bone-derived protein hormone osteocalcin (OCL) [41]. These agonists have been found to elicit intracellular calcium accumulation in heterologous cells overexpressing GPR37 [24,41] and within peritoneal macrophages [24]. A complete list of the proposed ligands for GPR37 and GPR37L1 and their associated signaling pathways/physiological outputs are depicted in Table 1 and highlighted in Figure 2. Surprisingly, both NPD1 and OCL have been linked to improved recovery following ischemic stroke [35,42,43,44]. To date, the International Union of Basic and Clinical Pharmacology (IUPHAR) has not approved any of the aforementioned ligands, therefore GPR37 and GPR37 L1 remain classified as orphan receptors [45].

### 1.3. N-Terminal Cleavage

Both GPR37 and GPR37 L1 undergo constitutive a metalloproteinase cleavage of their N-terminus [38,46,47]. This phenomenon of ectodomain shedding has been extensively described for the following GPCRs: protease-activated receptors (PARs) [48] and adhesion receptors [49]. For GPR37 L1, the cleavage of the N-terminus was linked to a loss of constitutive (ligand independent) receptor signaling through Gαs [38]. These data were assessed within heterologous cells overexpressing a tagged receptor construct, therefore the physiological relevance of this effect remains unclear. For GPR37, the signaling effects being mediated by the proteolytic processing of the receptors N-terminus were not assessed. It has, however, been established that its ectodomain cleavage is specifically modulated by the metalloproteinase’s ADAM10 and furin [47]. Interestingly, the proteolytically processed species of both GPR37 and GPR37L1 seem to dominate in vivo [38,47]. This suggests that under physiological conditions, both receptors are being rapidly processed into their proteolytically cleaved counterparts. Intriguingly, following middle the cerebral artery occlusion (MCAO), protein expression of a cleaved form of GPR37 (~40 kD) was found to be upregulated within the peri-infarct region [23].

The N-terminal cleavage of both GPR37 and GPR37 L1 by metalloproteases suggests that these enzymes may have the ability to control receptor activity and expression through post-translational modifications. To date, it remains unclear whether this mechanism can occur via receptor auto-catalytic mechanisms such as those employed by PARs and adhesion receptors. Under the GRAFS classification system, both GPR37 and GPR37 L1 belong to the β-group of rhodopsin receptors [50]. Interestingly, this type of modulation has been demonstrated to occur in other members of this group in a ligand-dependent manner: ET_B_ [51] and β1 adrenergic receptors [52,53]. The ability of proteolytic processing to alter GPCR signaling is a particularly interesting perspective given that matrix metalloproteases have been found to be upregulated [54] and modulate injury and recovery following ischemic stroke [55,56]. These enzymes are thought to play a complex role in stroke pathophysiology as they contribute to the injury response in the early stages and modulate critical recovery processes during later stages [56]. Although it remains unclear how proteolytic processing affects the function and signaling of GPR37 and GPR37L1, the N-terminal processing of these receptors likely reflects an important regulatory mechanism. Future studies should be aimed at characterizing which forms of the receptor are present/active in different physiological and pathophysiological conditions and whether the N-terminal cleavage represents a potential activation or inactivation mechanism.

## 2. Inflammatory Responses

Ischemic stroke evokes a robust inflammatory response that functions to modulate both acute and chronic responses to ischemic injury [57,58]. This response involves the activation of astrocytes [59,60,61], microglia [62,63,64], OPCs [65,66] as well as an influx of macrophages and other hematogenous cells across the blood vessel wall [67]. These cells are recruited to the injured zone through the coordination of cytokines, adhesion molecules and chemokines being secreted by activated cell types [67]. Early on, inflammation is thought to amplify damage induced by the ischemic lesion, however, it later on plays a critical role in setting the stage for tissue repair processes in more chronic stages.

### 2.1. Astrocytes

Ischemia leads to the accumulation of reactive astrocytes within the peri-infarct region hours following stroke onset. Following injury, these astrocytes undergo gliosis, become hypertrophic, and markedly upregulate a number of key proteins—one of the most prominent being the glial fibrillary acidic protein (GFAP). This process is acutely regulated and initially functions to form a barrier around damaged tissue and limit the spread of inflammatory signals, noxious chemokines and excitotoxic factors [61]. Interestingly, GPR37 has been found to be intricately involved in mediating astrogliosis following ischemic stroke. Acutely (24–72 h) following induction of MCAO, mice lacking GPR37 exhibited a larger infarct volume and substantially decreased activation of reactive astrocytes within the peri-infarct zone [23]. Within *Gpr37^-/-^* mice, the GPR37 promoter is linked to the LacZ reporter, allowing β-gal to be used as a proxy for GPR37 expression within these mice. Astrocytes within *Gpr37^-/-^* mice did not express detectable levels of β-gal, thus suggesting that the GPR37-dependent modulation of astrogliosis likely occurs through paracrine-mediated—rather than direct—signaling effects.

At later timepoints following injury, reactive astrocytes contribute significantly to the formation of the glial scar, which functions as a physical and chemical barrier that prevents neurite regeneration and impedes functional recovery [59]. Ten days following stroke, *Gpr37*^-/-^ mice exhibited significantly reduced glial scar formation [68], potentially implying an enhanced regenerative response in these mice. Consistent with this notion, β-gal (proxy for GPR37) was found to be increased within Sox2 expressing neural stem/progenitor cells (NSPCs) which were significantly more abundant within the peri infarct region of *Gpr37*^-/-^ mice with respect to wild-type mice [23]. The final fate of these cells was not determined, but it is intriguing to think that, in the presence of diminished glial scar formation, these cells could undergo neuronal differentiation and enhance the regenerative capacity of *Gpr37*^-/-^ mice at longer timepoints following ischemic injury. Future studies which examine more chronic time points will utilize fate restricted mapping and incorporating behavioral outcomes will be key to understanding the functional consequences of these results.

In contrast to GPR37, GPR37 L1 is abundantly and almost exclusively expressed within astrocytes under physiological conditions [25]. One week following MCAO, the mRNA expression of GPR37 was found to be significantly upregulated within GFAP-positive astrocytes surrounding the peri-infarct zone with respect to the contralateral cortex [25]. Interestingly, although GPR37 L1 was found to be expressed within both OPCs and astrocytes in uninjured brains, its expression was only upregulated within reactive astrocytes following MCAO [25]. After chemical-induced ischemia, cultured hippocampal slices from mice lacking GPR37 L1 (*Gpr37 l1*^-/-^) displayed substantially increased neuronal death, suggesting that GPR37 L1 functions in a neuroprotective manner following ischemia ex vivo [25]. Since the latter study did not directly assess stroke responses within *Gpr37 l1*^-/-^ mice, the functional consequences of disrupting this receptor in vivo remain unclear.

### 2.2. Microglia and Peripheral Macrophages

Shortly following the onset of stroke, microglia are activated and polarized into two distinct phenotypes—M1 and M2. M1 microglia release cytotoxic factors such as inflammatory cytokines and nitric oxide, whereas M2 microglia function in a more protective manner by removing cellular debris and promoting repair [62,69]. Interestingly, *Gpr37^-/-^* mice display an increase in the number of Iba1 positive microglia and macrophages 24 h following MCAO. These mice also exhibited increased an mRNA expression of M1 microglia markers and an upregulation of the following inflammatory chemokines: monocyte chemoattractant protein 1 (MCP-1) and macrophage inflammatory protein 1a (MIP-1a) [68]. These secreted factors function to augment inflammation and immune-cell infiltration into the ischemic cortex by attracting more immune cells into the ischemic region, thereby further contributing to pathologic inflammation [69].

Since Iba1 detects both microglia and infiltrating peripheral macrophages, it remains unclear whether the loss of GPR37 signaling leads to a more substantial increase in resident microglia or infiltrating macrophages. A recent study demonstrated that GPR37 is expressed within macrophages but not microglia, where its activation by TX41A and NPD1 triggers calcium-induced phagocytosis [24]. Future studies are needed to assess the contribution of macrophage-expressed GPR37 in resolving inflammation and repair following stroke. The contribution of GPR37 L1 in microglial polarization and macrophage dynamics has not been investigated, but the combination of GPR37 L1′s high expression profile within astrocytes and the ability for astrocytic factors to modulate microglial responses following injury [70,71] could suggest a potential regulatory role for GPR37 L1 in the modulation of microglial responses following ischemic stroke.

### 2.3. Oligodendrocyte Progenitor Cells

**Figure 3:**
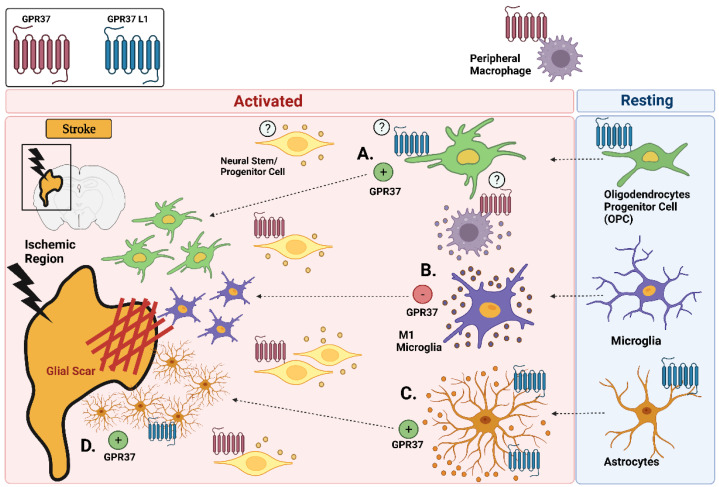
Potential roles for GPR37 and GPR37 L1 in modulating glial cell activation and recruitment following ischemic injury. Key cell types expressing GPR37 and GPR37 L1 are highlighted: (**A**) GPR37 stimulates the activation of OPCs; (**B**) GPR37 inhibits the production of M1 microglia and the secretion of inflammatory cytokines; (**C**) GPR37 stimulates the formation of reactive astrocytes; and (**D**) GPR37 stimulates the formation of the glial scar. (Image created with BioRender.com (accessed on 28 February 2022).

In response to ischemic injury, OPCs expressing the neural/glial antigen 2 (NG2) undergo injury-induced activation [72], become hypertrophic, and proliferate within the peri-infarct zone [66,73]. In *Gpr37*^-/-^ mice, these cells were found to express significantly less NG2 three days following MCAO [23]. These findings are consistent with attenuated astrocyte activation within the peri-infarct region of *Gpr37*^-/-^ mice. Interestingly, GPR37 was not expressed by OPCs following MCAO [23]. This is consistent with brain transcriptomic data [19] and suggests that GPR37 signaling may be mediating its effects on gliosis and the glial microenvironment through non-direct paracrine-mediated signaling effects. One potential hypothesis is that GPR37 signaling within NSPCs affects the secretion of soluble factors capable of modulating gliosis and tissue repair. Indeed, multiple mechanisms of communication exist between NSPCs and neighboring cells to control numerous aspects of the injury microenvironment [74,75,76]. In contrast to GPR37, GPR37 L1 is highly expressed within OPCs [19,25]. Although its expression was not found to increase following MCAO [25], it cannot be ruled out that the direct activation of GPR37 L1 within OPCs could be involved in modulating the activation profile of these cells following ischemic injury. Figure 3 provides a summary of inflammatory responses being mediated by GPR37 GPR37L1 following ischemic stroke.

### 2.4. Neural Stem and Progenitor Cells

Within the adult mammalian brain there are two key neurogenic regions: the SVZ and the subgranular zone of the dentate gyrus (SGZ). Quiescent and activated NSPCs within these two distinct regions function to generate new neurons within the adult brain. Ischemic stroke in rodents leads to an increase in neurogenesis within the SVZ, consequently triggering the migration of NSPC-derived neuroblasts from the SVZ to the boundary of the ischemic injury [77,78,79,80]. Notably, stroke-induced neurogenesis has also been demonstrated within the human brain [81,82,83]. A recent study demonstrated that GPR37 is expressed within NSPCs where it is required for Wnt-dependent neurogenesis [22]. Moreover, NSPCs appear to accumulate around the peri-infarct and core region of *Gpr37*^-/-^ mice three days following MCAO [23]. The latter potentially suggests an inhibitory role of GPR37 signaling on NSPC dynamics (Figure 3). Additional studies are necessary to better characterize what effects GPR37 signaling imparts on both NSPC proliferation and differentiation. Interestingly, a recent study identified that GPR37 L1 is highly expressed within quiescent neural stem cells within the SVZ [26]. Future studies aimed at understanding the functional relevance of GPR37 L1 signaling within these cells and their regulation following ischemic injury would be beneficial.

## 3. Other Regulatory Mechanisms

### 3.1. Excitotoxicity

During the early stages of ischemic stroke, glutamate accumulation within the extracellular space occurs as a result of ion pump failure and the breakdown of various glutamate reuptake mechanisms [84]. Consequently, this overabundance of glutamate leads to the overstimulation of AMPA and NMDA receptors resulting in an influx of calcium which triggers cell death [84]. Under physiological conditions, mice lacking GPR37 exhibit decreased levels of glutamate receptor subunits, total and phosphorylated AMPA receptor subunit 1 (GluA1), and NMDA receptor subunit 2B (GluN2B) within the striatum [85]. These expression level differences suggest that in the uninjured brain, *Gpr37*^-/-^ mice may exhibit the decreased membrane availability of AMPARs and NMDARs. It is unknown whether these differences persist in the cortex and to what extent they may be modulated by ischemic injury. The same study also demonstrated that *Gpr37*^-/-^ mice exhibit increased levels of striatal GABA following intrastriatal lesioning [85]. Given the potential role for inhibitory GABAergic interneurons in modulating the brain’s plasticity and repair following stroke [86,87], the ability of GPR37 to modulate this response would be an intriguing direction for further investigation. Future studies are needed to fully understand the role of GPR37 in modulating both inhibitory and excitatory signals which regulate excitotoxity following stroke.

The molecular mechanisms underlying GPR37L1 activation within astrocytes and its potential contribution to regulating glutaminergic signaling remain poorly understood. Within hippocampal slice preparations, GPR37 L1-mediated signaling (via prosaptide, 10 μM) inhibited astrocytic glutamate transporters and reduced neuronal NMDA signaling during prolonged stimulation [25]. However, the global deletion of GPR37 L1 did not alter the mRNA abundance of key glutamate transporters GLT-1 and GLAST within the hippocampus of mice at 14 or 40 days of age [25]. The latter result does not rule out the possibility that differences in the expression level of astrocytic transporters may exist in other brain regions or become apparent in *Gpr37 l1^-/-^* mice following ischemic injury. Since GPR37 L1 was not found to be expressed within neurons, the inhibitory effect the receptor evoked on neuronal NMDA currents involves an indirect modulatory mechanism via which astrocytic signaling functions to inhibit calcium-induced neurotoxicity following ischemia [25]. Additional studies are needed to further clarify this mechanism.

### 3.2. Cell Death Pathways

Cellular repair following stroke depends on a delicate balance between restorative and destructive pathways following ischemic injury [88]. If more damage than repair occurs, cell death mechanisms including apoptosis, necrosis, and autophagy may be employed [88]. During the acute phases of MCAO in mice, GPR37 functions to inhibit cell death [68] and the presence of GPR37 L1 has been demonstrated be neuroprotective within hippocampal slices exposed to chemical ischemia [25]. Although the potential mechanisms mediating neuroprotection via GPR37 L1 remain unresolved, GPR37 has been shown to reduce both apoptosis and autophagy within the peri infarct region hours following MCAO. More specifically, the loss of GPR37 was associated with increases in the expression of the proapoptotic proteins Bax and activated caspase-3 and decreases in the antiapoptotic protein Bcl-2. Moreover, *Gpr37^-/-^* mice exhibited significant increases in the cellular levels of the pro-autophagic Beclin-1 protein as well as the increased inhibition of the anti-autophagic mTOR pathway [68]. These data are consistent with those of a prior work suggesting a role for GPR37 in regulating autophagy [89].

In recent years, studies highlighting the functional relevance of autophagy in ischemia-induced neuronal damage have indicated the opposite results with some studies citing protective effects against neuronal injury [90,91] and others deleterious [92,93,94]. It is important to note that the data collected by McCrary et al. [68] analyzed markers for apoptosis and autophagy using peri-infarct tissue and therefore reflects a summary of all apoptotic and autophagic cell death outcomes. The interpretation of these data is thus limited with respect to our understanding of GPR37-mediated cell type specific responses. Moreover, these studies only assessed the acute regulation of these pathways, therefore additional timepoints would help provide a more comprehensive picture of the regulatory effects of GPR37 on both apoptotic and autophagic pathways following ischemia.

### 3.3. Hypertension

Hypertension is currently the most prevalent risk factor for stroke and has been reported in more than 60% of stroke patients [95]. A number of studies have now demonstrated that GPR37 L1 plays a critical role in maintaining blood pressure homeostasis and may represent a unique target for the treatment of hypertension [96,97,98]. A study examining the expression profiles of patients with myocardial infarction found that GPR37 L1 was one of the top genes associated with heart failure and that the loss of GPR37 L1 in mice resulted in significantly higher blood pressure levels [96]. Although GPR37 L1 was found to be expressed within the myocardium of patients suffering from heart failure, its expression profile within mouse cardiovascular tissue under normal physiological conditions could not be detected at the protein level [97]. GPR37 L1 instead was found to be substantially expressed within key cardiovascular regulating regions of the CNS including the nucleus of the solitary tract, caudal and rostral ventrolateral medulla and the A5 nucleus [97]. This may suggest that, under physiological (non-pathological) conditions, many of the effects which GPR37 L1 exert on blood pressure regulation occur through central regulatory mechanisms within the CNS as opposed to peripheral cardiovascular control.

Interestingly, the regulatory mechanisms via which GPR37 L1 controls blood pressure are sexually dimorphic, resulting in a female-specific increase in systolic, diastolic, and mean arterial pressure [97]. However, when male and female mice were challenged with an AngII infusion, only male *Gpr37 l1*^-/-^ mice displayed evidence of heart failure whereas female *Gpr37 l1*^-/-^ mice were protected [97]. Therefore, in females, GPR37 L1 is intricately involved in baseline blood pressure parameters, whereas in males, it appears to be more critical for regulating cardiovascular compensatory responses. Further studies revealed that both male and female *Gpr37 l1*^-/-^ mice exhibit a generally reduced sympathetic vasomotor tone which is likely linked to alterations in the autonomic regulation of blood pressure and responses to stressful stimulus in these mice is in fact sexually dimorphic [97]. These studies prompt some interesting observations with respect to potential roles for GPR37 and GPR37 L1 in ischemic stroke: (1) studies performing MCAO in *Gpr37 l1*^-/-^ mice are needed to further understand this receptor’s contribution; (2) all stroke studies examining the functional relevance of GPR37 following MCAO have only been done using male mice and should be expanded to include female mice.

### 3.4. Interacting Partners

GPR37 physically interacts and forms heterodimers with D2R [31,99], A_2A_R [100,101], and serotonin 5-HT4d receptors [101]. GPR37 L1 has also been proposed to physically interact with D2R within live cells [99]. The only interaction which has been validated in native tissue thus far is the GPR37/A_2A_ heterodimer [100]. Interestingly, all of the aforementioned receptors have been proposed to have differential roles in modulating stroke pathophysiology. In the uninjured brain, D2R receptors are almost exclusively expressed within neuronal cells. Ischemic injury leads to an increase in the expression of D2R receptors within activated astrocytes [102], microglia [9], as well as peripherally derived macrophages [9]. The expression of 5-HT4 receptors has also been noted within peripheral macrophages following stroke [103,104]. Although GPR37 is not expressed on reactive astrocytes, the loss of the receptor impairs astrocyte activation by mechanisms which are not fully understood. Since GPR37 is expressed within peripheral macrophages, the co-expression profile of GPR37/D2R and GPR37/5-HT4 within these cells could potentially suggest some physiological relevance for these heterodimers in modulating macrophage dynamics. Likewise, a potential for GPR37 L1/D2R heterodimers may exist within astrocytes following ischemia. A_2A_R is expressed within NSPCs and its activation stimulates cellular proliferation [105]. This implies that there may exist a functional role for GPR37/A_2A_R heterodimers in modulating the proliferation and differentiation of NSPCs following ischemic injury. Further studies would be needed to validate the formation of these interactions in vivo.

## 4. Conclusions

Amidst numerous discrepancies associated with current proposed ligands, GPR37 and GPR37 L1 remain classified as orphan receptors. Both receptors are expressed on distinct glial cell populations which participate in modulating the injury response in both the acute and chronic phases of ischemic stroke. GPR37 is expressed within NSPCs and peripheral macrophages whereas GPR37 L1 is predominantly expressed in astrocytes and OPCs. Following ischemic injury, glial cells become reactive and communicate with one another by secreting various factors into the injury microenvironment. Cellular communication via direct and paracrine mechanisms thus adds an additional layer of complexity to disentangling the precise contribution of GPR37 and GPR37 L1 signaling in ischemic stroke. Moreover, only two studies have directly evaluated the effects of MCAO in *Gpr37^-/-^* mice and there are currently no studies which have evaluated the functional consequences of ablating GPR37 L1 in MCAO. Notwithstanding, GPR37 and GPR37 L1 are emerging as key regulators of inflammation, excitotoxicity, and cell death following ischemic injury. Additional studies are needed to further elucidate the precise mechanisms via which these receptors regulate gliosis and modulate repair following stroke.

## Figures and Tables

**Table 1:** Proposed ligands for GPR37 and GPR37 L1.

Ligand	Receptor	Mechanisms and Signaling Pathways
Head Activator 2 nM	GPR37	Receptor internalization within heterologous cells [29] Calcium stimulation via Ga16/aequorin assay [29] Calcium-mediated NFAT transcription [30] Inhibition of cAMP accumulation [30]
Prosaposin/TX14A 100 nM–1 µM	GPR37 GPR37 L1	Receptor internalization within heterologous cells [34] ERK1/2 phosphorylation [34], GTPγS accumulation [34], inhibition of cAMP within HEK293T cells [34] ERK1/2 phosphorylation and protection from oxidative stress within primary astrocytes [34] Macrophage phagocytosis of zymosan particles via calcium signaling [24]
Neuroprotection D1 30 nM	GPR37	Increase in intracellular calcium levels within HEK292T cells [24] Increase in intracellular calcium levels coupled with the active phagocytosis of zymosan particles within peritoneal macrophages [24]
Osteocalcin 20 nM	GPR37	Increase in intracellular calcium levels in HEK293-GCamP6s cells [41] β-arrestin recruitment using a PRESTO-Tango GPCR assay system in HTLA cells [41] Inhibition of cAMP and stimulation of ERK phosphorylation within HEK293 cells overexpressing GPR37 [41]
